# Effects of a Non-Conservative Sequence on the Properties of β-glucuronidase from *Aspergillus terreus* Li-20

**DOI:** 10.1371/journal.pone.0030998

**Published:** 2012-02-07

**Authors:** Yanli Liu, Jie Huangfu, Feng Qi, Imdad Kaleem, Wenwen E, Chun Li

**Affiliations:** 1 School of Chemical Engineering and Technology, Tianjin University, Nankai District, Tianjin, People's Republic of China; 2 School of Life Science, Beijing Institute of Technology, Haidian District, Beijing, People's Republic of China; Russian Academy of Sciences, Institute for Biological Instrumentation, Russian Federation

## Abstract

We cloned the β-glucuronidase gene (*At*GUS) from *Aspergillus terreus* Li-20 encoding 657 amino acids (aa), which can transform glycyrrhizin into glycyrrhetinic acid monoglucuronide (GAMG) and glycyrrhetinic acid (GA). Based on sequence alignment, the C-terminal non-conservative sequence showed low identity with those of other species; thus, the partial sequence *At*GUS(-3t) (1–592 aa) was amplified to determine the effects of the non-conservative sequence on the enzymatic properties. *At*GUS and *At*GUS(-3t) were expressed in *E. coli* BL21, producing *At*GUS-E and *At*GUS(-3t)-E, respectively. At the similar optimum temperature (55°C) and pH (*At*GUS-E, 6.6; *At*GUS(-3t)-E, 7.0) conditions, the thermal stability of *At*GUS(-3t)-E was enhanced at 65°C, and the metal ions Co^2+^, Ca^2+^ and Ni^2+^ showed opposite effects on *At*GUS-E and *At*GUS(-3t)-E, respectively. Furthermore, *K*m of *At*GUS(-3t)-E (1.95 mM) was just nearly one-seventh that of *At*GUS-E (12.9 mM), whereas the catalytic efficiency of *At*GUS(-3t)-E was 3.2 fold higher than that of *At*GUS-E (7.16 vs. 2.24 mM s^−1^), revealing that the truncation of non-conservative sequence can significantly improve the catalytic efficiency of *At*GUS. Conformational analysis illustrated significant difference in the secondary structure between *At*GUS-E and *At*GUS(-3t)-E by circular dichroism (CD). The results showed that the truncation of the non-conservative sequence could preferably alter and influence the stability and catalytic efficiency of enzyme.

## Introduction

**Figure 1:**
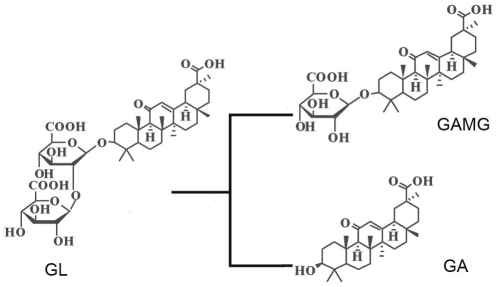
Biotransformation of GL directly into GAMG and GA. GL can be converted into GAMG by hydrolyzing the distal glycosidic bond and GA by the removal of two molecules of glucuronide simultaneously.

Glycyrrhizin (GL), the main constituent of licorice extract (*Glycyrrhiza glabra*), is a natural edulcorant as well as an important ingredient of traditional Chinese medicine [1], [2], [3]. By hydrolyzing one or two distal glucuronides, GL can be transformed into glycyrrhetinic acid monoglucuronide (GAMG) or glycyrrhetinic acid (GA) (**Figure 1**). As an important derivative of GL, GAMG displayed stronger physiological functions than GL such as anti-viral, anti-inflammatory, anti-tumor functions, and so on; and it is also 1000-fold sweeter than saccharose [4]. On the other hand, GA is the bioactive substance of GL well known for its pharmacological features [4], [5], [6]. The research on GL biotransformation catalyzed by β-glucuronidase (GUS, EC 3.2.1.31) was reported mainly in animal tissues such as duck [7] and human [8], whereas studies on GL biotransformation in fungal species are few [9]. In our previous work, a fungal strain, *Aspergillus terreus* Li-20 was screened, which can use GL as a carbon source and produce GAMG and GA after catalysis by β-glucuronidase (*At*GUS). The main disadvantages of *At*GUS were low enzyme productivity, low catalytic efficiency, and pathogenicity, which rendered it unsafe for use in the food and medical industries.

To overcome disadvantages of a natural enzyme, many methods was applied to obtain artificial evolution enzymes, and this approach is not only faster than natural evolution but also provides a deeper understanding of enzyme evolution. Several methods of designing new enzymes are available, and gene sequence truncation is also investigated for its effects on enzymatic properties. The non-conservative N-terminal domain of the protein phosphatase1 (PP1), with 1–8 residues deleted, showed higher sensitivity to three substrates and influenced the structure and properties of PP1 [10], whereas the truncation of the C-terminal region improved the thermal stability of endo-β-glucanase from *Bacillus subtilis* JA18 [11]. However, the loss of the C-terminal regulatory domain resulted in a loss of the ability to catalyze the aldol reaction [12].

With development of molecular biology and bioinformatics characterization, an increasing number of sequence data have been cloned and applied in the biotransformation industry. Bioinformatics characterization from the National Center for Biotechnology Information (NCBI) showed that most β-glucuronidases belong to the glycoside hydrolase family (GHF) 2, and all of them consist of sugar-binding, immunoglobulin-like β-sandwich, and TIM barrel domains (triosephosphate isomerase, TIM) [13], [14], [15]. The TIM barrel domain, which is one of the most common catalytic domains, is adopted by about 10% of the enzymes; thus, sequence modification inside or outside the domain to improve the enzymatic property and determine the catalytic mechanism was reported in many studies. The site-directed mutagenesis of seven amino acids (aa) in the TIM barrel domain was performed to investigate the importance of the residue in the catalysis of an exo-β-d-glucosaminidase from *Trichodema reesei* [16]. Heparanase is an endo-β-d-glucuronidase, and its C-terminal region, which is not an integral part of the TIM barrel domain, is essential for the enzymatic activity and secretion of heparanase [17].

Although β-glucuronidases from many species have been registered in Genbank, only a few genes have been published for GL biotransformation [18]. Three fungal strains, namely, *A. terreus* Li-20, *P. purpurogenum* Li-3, and *A. ustus* Li-62, were screened in our previous studies and represented three modes of GL biotransformation: (1) GL→GA+GAMG; (2) GL→GAMG; and (3) GL→GA [9]. The three β-glucuronidase genes were cloned in our laboratory, and the aa sequence alignment showed that the β-glucuronidase from *A. terreus* Li-20 (*At*GUS) was quite different from the other two β-glucuronidases(*P*GUS and *Au*GUS) in C-terminal non-conservative sequence. *At*GUS can hydrolyze GL into two products; thus, the different modes of GL biotransformation of the β-glucuronidases from the other two fungi may be related to the natural evolution in the sequence. In the present research, *At*gus and the partial sequence [*At*gus(-3t)] without C-terminal non-conservative sequence behind the TIM barrel domain were amplified in order to investigate effects of non-conservative sequence on enzymatic property.

## Materials and Methods

### Ethics Statement

No specific permits were required for the described field studies. No specific permissions were required for these locations/activities. No location is privately-owned or protected in any way. The field studies did not involve endangered or protected species.

### Strains, plasmids, and culture conditions

In our previous work, *A. terreus* Li-20 was isolated and screened from a *G. glabra* planting field in Shihizi, Xinjiang. It was incubated in 100 ml liquid Czapek's medium in a 500 ml Erlenmeyer flask at 30°C and placed in a shaker incubator at 170 rpm.

*Escerichia coli* DH5α and *E. coli* BL21 were used as hosts for plasmid amplification and expression, respectively. The plasmids pMD19-T (TaKaRa, Japan) and pET28a (+) (Invitrogen, U.S.) were used as vectors. The recombinant cells were inoculated in a lysogeny broth (LB) medium with kanamycin (50 mM) and operated at 37°C for 3 h. The recombinant protein was induced by adding 0.4 mM isopropyl-β-D-thiogalactopyranoside (IPTG).

### Chemicals and reagents

GL was purchased from Xinjiang Tianshan Pharmaceutical Co. (China). GA was purchased from Sigma Chemical Co. (U.S.), whereas GAMG was obtained from the Nanjing University of Technology, China. Methanol was of chromatographic grade. All other chemicals used were of analytical grade. The DL2000 marker and the protein low weight marker were purchased from TaKaRa, Japan.

### Gene coloning and vector construction

**Figure 2:**
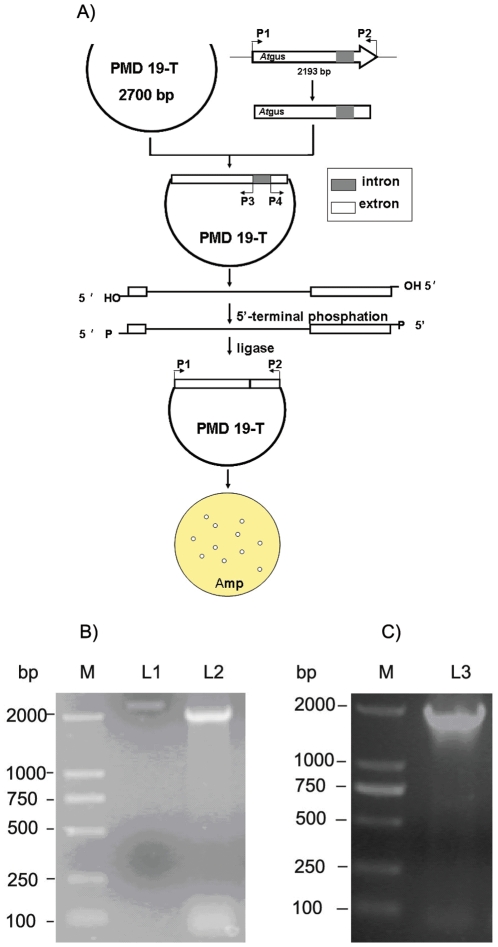
Intron deletion of *At*gus (A) and electrophoresis of the target sequence(B and C). M: marker, DL2000; L1: the target gene with intron; L2: the full length of the gene without intron(*At*gus); L3: the target gene without C-terminal non-conservative sequence[*At*gus(-3t)].

An intron in an *At*GUS genomic sequence was removed via three-step polymerase chain reaction (PCR) to express the gene in *E. coli* BL21 (**Figure 2A**). According to the database of *A. terreus* NIH2624, a primer set containing P1(5′-CCGTACgTAATGCTGAAGCCCCGACAAACACCTT-3′) and P2(5′-CATGCGGCCGCTTAAGCGCCAAATAGGAAGTATAGT-3′) was designed to obtain the sequence with an intron from the *A. terreus* Li-20 genome under the following conditions: 94°C for 10 min, 30 cycles of 94°C for 1 min, 58°C for 1 min, 72°C for 2 min, and a final extension at 72°C for 10 min with Ex Tag (TaKaRa, Japan). After ligated into PMD19-T, a primer set containing P3(5′-CACTCCACCGTGTTTTCAATGTATGAGCTGCAGC-3′) and P4(5′-CCGGCTTCGCAGCTATGTGTCTTGAGCATC-3′) was used for the second PCR, and the reaction was performed by Pfu polymerase (Shenggong, China) under the following conditions: 94°C for 10 min, 30 cycles of 94°C for 1 min, 55°C for 1 min, and 72°C for 5 min. The fragment amplified in the second PCR was ligated by T4 DNA ligase after a terminal phosphation with T4 polynucleotide kinase (Takara, Japan), and the positive clones were screened in an LB plate with 100 µg/mL ampicillin. The primer set containing P1 and P5(CATGCGGCCGCTTAACTCCACCGTGTTTTCAATGTATG-3′) was used for *At*GUS(-3t) under the same conditions as those of P3 and P4.

### Purification of *At*GUS-E and *At*GUS(-3t)-E

After IPTG introduction and the ultrasonication of the recombinant *E. coli* BL21 cells, supernatant was brought to 70% saturation with (NH4)_2_SO_4_ and stored overnight at 4°C, and then again centrifuged. The enzymes expressed by pET28a(+) vector were fused to an N-terminal six-histidine tag and purified via nickel chelate affinity chromatography (GE, U.S.), which was eluted with 150 mM imidazol. The quality of the purified protein was evaluated using sodium dodecyl sulfate polyacrylamide gel electrophoresis and coomassie blue staining.

### HPLC for analysis of GL, GAMG, and GA

GL, GAMG, and GA concentrations were measured via reverse-phase high performance liquid chromatography (HPLC) on a C18 column (4.6 mm×250 mm, 5 µm particle size, Kromasil) at 40°C. The sample (injection volume, 10 µl) was separated with a mobile phase consisting of 6% acetic acid/methanol (19∶81 v/v), and the elution was monitored via ultraviolet detection at 254 nm. The GL, GAMG, and GA amounts were calculated from the standard curve of the peak area and concentration.

### Determination of pH and temperature profiles

The activity of β-glucuronidase was determined using GL as the substrate. The reaction mixture consisted of the enzyme and substrate (2 g/L GL) at a 1∶4 (v/v) ratio.

50 Mm Na_2_HPO_4_-citric acid buffer at pH 4.0–8.0 was used for determine the pH effects of the enzyme. The catalytic activity of the enzyme was examined at 30 to 70°C in 50 mM Na_2_HPO_4_-citric acid buffer (pH 7.0). The enzyme activity under the optimal temperature and pH was defined as 100%.

The temperature stability of the enzyme was determined by incubating the enzyme samples at different temperatures (45, 55, 65, and 75°C) for 15, 30, 45, 60, and 120 min at optimum pH without the substrate GL, and the residual activity was determined at the optimum temperature.

### Determination of metal ions profiles

The effect of several metal ions on the activity of *At*GUS(-3t)-E and *At*GUS-E was investigated. The enzyme activity was determined in the reaction mixture consisting of K^+^(KCl), Na^+^(NaCl), Mg^2+^(MgCl_2_), Mn^2+^(MnCl_2_), Co^2+^(CoCl_2_), Ca^2+^(CaCl_2_), Ni^2+^(NiSO_4_), Cu^2+^(CuSO_4_), and Al^3+^(AlCl_3_) ions at final concentrations of 1 and 5 mM. The enzyme activity was subsequently determined at the optimum temperature after incubation for 30 min.

### Determination of kinetic parameters

Different concentrations of the substrate GL, ranging from 0.375 to 4 mM, were prepared to determine the kinetic constants. The catalytic reactions were continuously monitored, and the initial velocities were fitted to the Michaelis-Menten equation using the Origin 7.5 software (OriginLab). The values of the Michaelis-Menten constant (*K*m), maximal velocity (*V*max), catalytic turnover rate (*K*cat), and catalytic efficiency (*K*cat/*K*m) were evaluated.

### Analysis of Circular dichroism specta (CD)

Far-UV Circular dichroism (CD) spectra were recorded at 25°C in the range from 190 to 260 nm with a spectral resolution of 0.2 nm using a Jasco J-715 spectropolarimeter. The scan speed was 100 nm/min and the response time was 0.125 s with a bandwidth of 1 nm. Quartz cells with an optical path of 0.1 cm were used. Typically, scans were accumulated and subsequently averaged. The spectra were corrected for the corresponding protein-free control.

### Modelling of protein structure

The protein three-dimensional structural was modeled by modeler 9v7 to analyze three domains of the protein.

## Results

### Gene cloning and sequencing analysis

The 2,193 bp product was amplified and sequenced using a genomic template (**Figure 2B**), and its 219 bp intron was analyzed by NCBI. After a three-step PCR, the full encoding sequence was cloned. The results show that the open reading frame of this gene was 1,974 bp (**Figure 2B**), which encodes for 657 aa.

**Figure 3:**
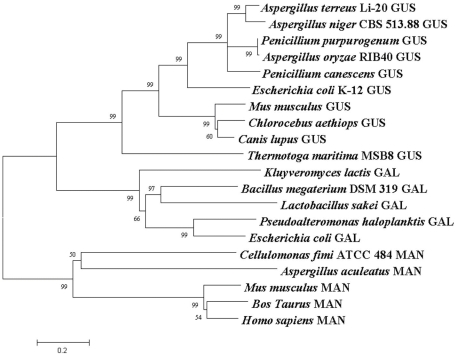
Phylogenetic tree of target protein with GHF2 β-glucuronidase (GUS), β-galactosidase (GAL), β-mannosidase (MAN). The scale corresponds to a genetic distance of 0.2 substitution per position (20% difference). *Aspergillus niger* CBS 513.88 GUS: XP_001388566; *Penicillium purpurogenum* GUS: ABU68712; *Aspergillus oryzae* RIB40 GUS: XP_001825002; *Penicillium canescens* GUS: AAV91787; *Escherichia coli* K-12 GUS: AAC74689; *Mus musculus* GUS: AAA37696; *Chlorocebus aethiops* GUS: AAC34593; *Canis lupus* GUS: AAC48809; *Thermotoga maritima* MSB8 GUS: AAD36143; *Kluyveromyces lactis* GAL: AAA35265; *Bacillus megaterium* DSM 319 GAL: CAA04267; *Lactobacillus sakei* GAL: CAA57730; *Pseudoalteromonas haloplanktis* GAL: CAA10470; *Escherichia coli* GAL: AAA24053; *Cellulomonas fimi* ATCC 484 MAN: AAD42775; *Aspergillus aculeatus* MAN: BAA29029; *Mus musculus* MAN: AAK18177; *Bos Taurus* MAN: AAC48460; *Homo sapiens* MAN: AAC39573.

The conserved domain database (CDD) was performed to analyze domains of *At*GUS, and there were sugar-binding domain, immunoglobulin-like beta-sandwich domain, and TIM barrel domains in it which all belonged to glycoside hydrolase family (GHF) 2. GHF 2 comprised β-galactosidase (EC 3.2.1.23), β-mannosidase (EC 3.2.1.25), and β-glucuronidase (EC 3.2.1.31), so the phylogenetic tree was constructed according to it (**Figure 3**). It showed that the gene cloned was a β-glucuronidase gene named *At*GUS (Genbank accession No. **JF894133**), which was found very similar to *P*GUS(Genbank accession No. **EU095019**) from *P. purpurogenum* Li-3 and *Au*GUS (Genbank accession No. **JN247805**) from *A. ustus* Li-62, especially in the sugar-binding, immunoglobulin-like beta-sandwich, and TIM barrel domain (**Table 1**). The obvious difference among them lied in the non-conservative sequence of the C-terminal behind the TIM barrel domain which may result in the difference enzymatic properties. Therefore, the 1–1,776 bp segment, named *At*gus(-3t), was amplified in the present study (**Figure 2C**) to determine the effects of the non-conservative sequence on β-glucuronidase.

**Table 1:** Domains analysis of *At*GUS, *P*GUS and *Au*GUS.

protein	Sugar-binding domain		Immunoglobulin-like beta-sandwich domain		TIM barrel domain	
	Sequence (aa)	E-value^a^	Sequence (aa)	E-value^b^	Sequence (aa)	E-value^c^
*At*GUS	9–180	1.60e^−43^	182–276	1.50e^−8^	278–592	2.30e^−117^
*P*GUS	9–179	2.80e^−45^	181–274	1.90e^−11^	276–594	3.10e^−120^
*Au*GUS	48–219	1.40e^−46^	222–316	1.10e^−13^	318–638	2.20e^−114^

a the E value was obtained by alignment with pfam02837, glycosyl hydrolase family 2, sugar binding domain;

b the E value was obtained by alignment with pfam00703, glycosyl hydrolase family 2, immunoglobulin-like beta-sandwich domain;

c the E value was obtained by alignment with pfam02836, glycosyl hydrolase family 2, TIM barrel domain.

### Protein expression and purification

**Figure 4:**
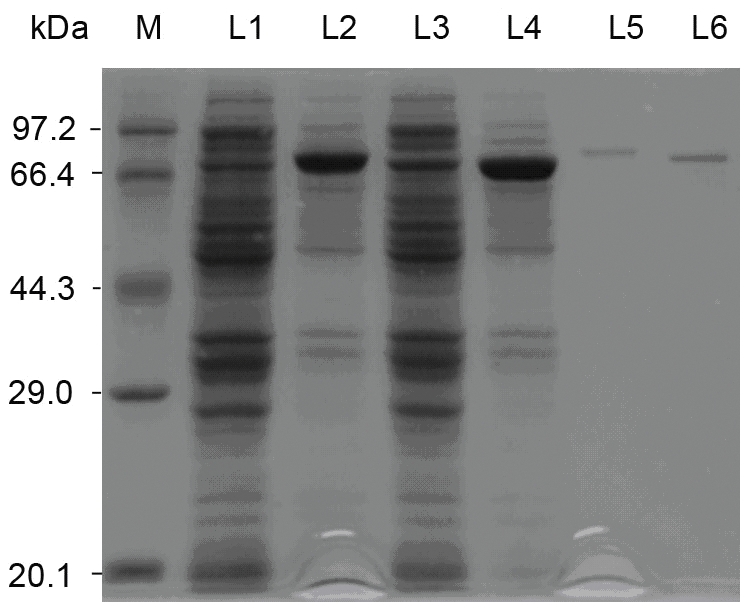
SDS-PAGE of recombinant protein. M: protein low weight marker; L1 and L3: whole cell of non-induced pET-28a(+)-*At*gus/BL21 and pET-28a(+)-*At*gus(-3t)/BL21; L2 and L4: whole cell of pET-28a(+)-*At*gus/BL21 and pET-28a(+)-*At*gus(-3t)/BL21 after induced with 0.4 mM IPTG; L5 and L6: purified *At*GUS-E and *At*GUS(-3t)-E.

Both pET28a(+)-AtGUS and pET28a(+)-AtGUS(-3t) were constructed and transformed into the *E. coli* BL21 strain, and the recombinant proteins AtGUS-E and AtGUS(-3t)-E were successfully expressed (**Figure 4**). The induction condition for the optimum production of the two recombinant proteins was 20°C with 0.4 µM IPTG. Both AtGUS-E and AtGUS(-3t)-E were purified through Ni-NTA sepharose (**Figure 4**). The target protein was eluted with 150 mM imidazole. Furthermore, the concentrations of the soluble purified proteins of AtGUS-E and AtGUS(-3t)-E were determined as ∼7 and ∼12 mg/L, respectively. Both purified enzymes could hydrolyze GL into GAMG and GA.

### Effect of pH and temperature on enzyme activity and stability

**Figure 5:**
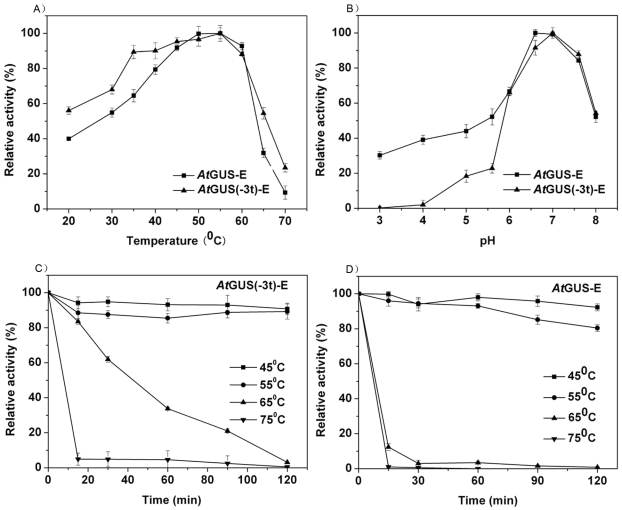
Enzymatic properties of *At*GUS-E and *At*GUS(-3t)-E. (A) the optimum pH, (B) the optimum temperature, and the thermal stability of *At*GUS(-3t)-E (C) and *At*GUS-E (D).

We investigated the enzymatic properties to determine the effect of the non-conserved sequence on the enzyme. The optimal pH for the bioconversion reaction by *At*GUS-E was 6.6, whereas that for *At*GUS(-3t)-E was 7.0 (**Figure 5A**).The optimal temperatures for *At*GUS-E and *At*GUS(-3t)-E were both 55°C (**Figure 5B**).

Enzyme thermal stability experiments showed that the enzymes remained more than 80% residual activity at 45°C and 55°C for 120 min heat treatment, respectively (**Figure 5C **and** 5D**). At 65°C, the residual activity of *At*GUS(-3t)-E remained almost 60% of enzymatic activity after 30 min heat treatment, which was comparatively higher than that of *At*GUS-E with less than 5% residual activity after the same treatment. At a higher temperature (75°C), both enzyme residual activity rapidly vanished, and within 15 min heat treatment, almost all enzymatic activity was lost.

### Effect of metal ions on enzymatic properties

The effect of various metal ions with different concentration gradients (from 1 to 5 mM final concentration) on the activities of *At*GUS(-3t)-E and *At*GUS-E was evaluated, and the results are presented in **Table 2**. The enzymatic activity assayed in the absence of metal ions was taken as 100%.

**Table 2:** Mental ion effect on *At*GUS-E and *At*GUS(-3t)-E.

Metal ions	Concentration(mM)	Relative activity (%)	
		*At*GUS-E	*At*GUS(-3t)-E
control	0	100	100
K^+^	1	102.22±1.45	99.02±2.65
	5	95.47±1.21	100.51±1.56
Na^+^	1	100.52±1.23	95.50±1.87
	5	86.16±1.85	86.41±2.15
Mg^2+^	1	126.99±2.15	118.63±2.56
	5	195.10±2.13	140.42±1.35
Mn^2+^	1	154.54±2.17	116.72±3.43
	5	187.63±3.12	200.15±1.87
Co^2+^	1	128.96±1.35	84.66±2.64
	5	99.26±1.43	162.96±3.65
Ca^2+^	1	144.64±3.12	84.10±3.12
	5	207.48±2.81	39.50±3.23
Ni^2+^	1	96.54±1.69	134.91±1.29
	5	60.03±2.98	178.16±4.16
Cu^2+^	1	24.37±3.89	41.18±3.85
	5	2.84±0.29	12.71±1.32
Al^3+^	1	136.47±2.51	107.09±1.34
	5	35.11±1.54	42.45±2.12

The effect of monovalent cations on the two enzymes was similar: the 1 and 5 mM K^+^ and 1 mM Na^+^ exhibited no obviously affecting effects on the activity of *At*GUS(-3t)-E and *At*GUS-E, while the 5 mM Na^+^ inhibited the enzymatic activity. The divalent cations Mg^2+^ and Mn^2+^ discretely promoted the activities of *At*GUS-E and *At*GUS(-3t)-E. With increasing concentration of Co^2+^, *At*GUS-E was firstly activated and then inhibited while *At*GUS(-3t)-E showed an inverse effect. Ca^2+^ and Ni^2+^ also exhibited opposite effects on the two enzymes. Ca^2+^ at 5 mM final concentration enhanced the activity of *At*GUS-E by 107% but inhibited *At*GUS(-3t)-E activity by 61%. In the presence of 5 mM Ni^2+^ buffer, *At*GUS(-3t)-E was increased by 78%, whereas *At*GUS-E was decreased by 40%. Cu^2+^ distinctively inhibited the enzyme activity, while Al^3+^ showed activation at 1 mM concentration and inhabitation at 5 mM concentration to both enzymes. These results reveal that K^+^, Na^+^, Mg^2+^, Mn^2+^, Cu^2+^, and Al^3+^ exhibited nearly similar effects on the activity of *At*GUS-E and *At*GUS(-3t)-E; however, Co^2+^, Ca^2+^, and Ni^2+^, showed opposite effects on both enzymes, respectively. The data reported here have been taken from three replicate samples from three independent experiments.

### Kinetic parameters

The reaction kinetics of *At*GUS-E and *At*GUS(-3t)-E were determined. The *V*max of the *At*GUS-E and *At*GUS(-3t)-E enzymes toward GL were calculated using Lineweaver-Burk plots (**Table 3**) and were determined as 1.84 and 0.97 µmol min^−1^ mg^−1^, respectively. The *K*m of the recombinant *At*GUS(-3t)-E was 1.95 mM, which was approximately one-seventh that of *At*GUS-E (12.9 mM), indicating that a higher affinity of *At*GUS(-3t)-E for GL than *At*GUS-E. In addition, the catalytic efficiency (*k*cat/*K*m) of *At*GUS(-3t)-E (7.16 mM s^−1^) was 3.2 folds higher than that of *At*GUS-E (2.24 mM s^−1^). The enzymatic activities were determined at different concentrations of the substrate GL from three independent experiments.

**Table 3:** Kinetic constants of recombinant protein *At*GUS-E and *At*GUS (-3t)-E.

Recombinant protein	*V*max (µmol min^−1^ mg^−1^)	*K*m (mM)	*K*cat (s^−1^)	*k*cat/*K*m (mM^−1^ s^−1^)
*At*GUS-E	1.84	12.9	29.0	2.24
*At*GUS(-3t)-E	0.97	1.95	13.6	7.16

### Structural characterization

**Figure 6:**
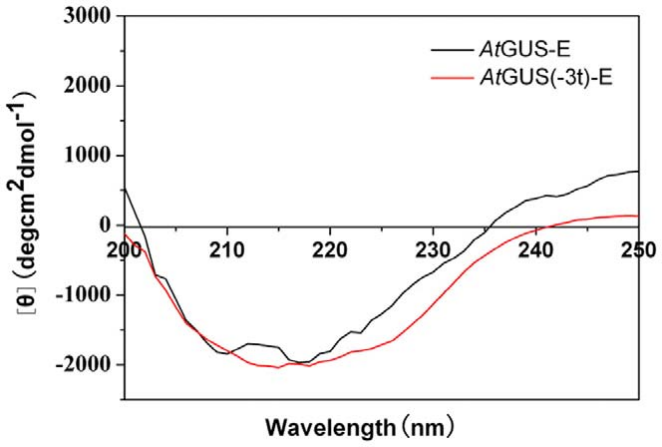
Far-UV spectra of *At*GUS-E and *At*GUS(-3t)-E. Far-UV spectra were recorded at 25°C in the range from 200 to 250 nm with a spectral resolution of 0.2 nm.

To determine the impact of the sequence truncation on the structure of the protein, a circular dichroism (CD) spectra was amplified. The far-UV spectra for *At*GUS-E and *At*GUS(-3t)-E have been presented in **Figure 6**. It illustrated that the curves exhibited significant difference between the two proteins. These results suggest that the secondary structure of *At*GUS-E has changed after deletion of non-conservative sequence.

## Discussion

The β-glucuronidase (GUS) gene was first cloned in 1987 [19], and in subsequent years, many GUS genes were cloned and registered in the GenBank. However, this gene has never been cloned for the hydrolysis research of GL into GAMG or/and GA, with more valuable merits. Based on CDD analysis, the three domains of the enzyme *At*GUS were well investigated, and the main aim of the present study is to modify the non-conservative sequence of *At*GUS and try to obtain an artificial evolution enzyme with better enzymatic properties.

**Figure 7:**
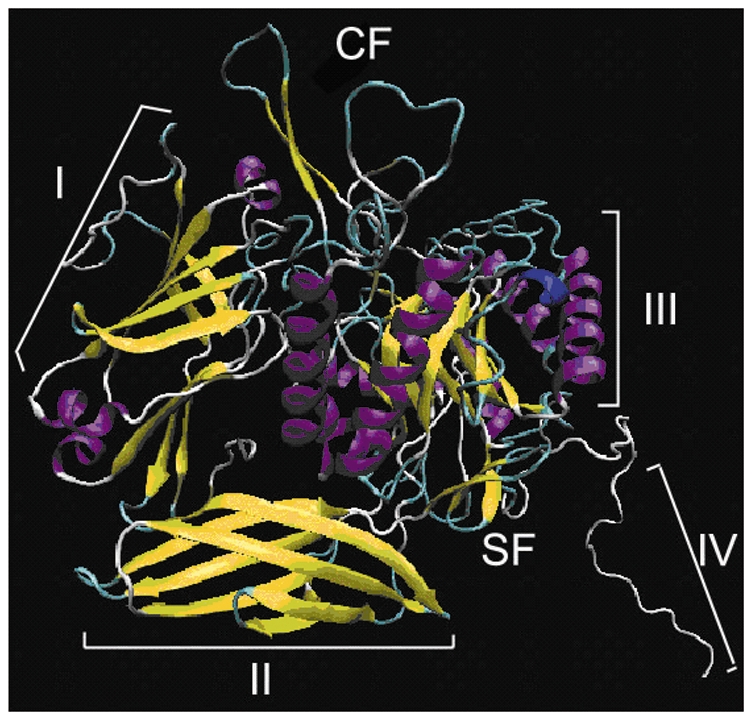
A three-dimensional model of β-glucuronidase(*At*GUS). I: sugar binding domain; II: immunoglobulin-like beta-sandwich domain; III: TIM barrel domain; IV: non-conservative domain; catalytic face and stability face were shown CF and SF for short.

The TIM barrel domain is a canonical (β/α)_8_-barrel composed of eight units, each of which consists of a β-strand and an α-helix [20]. There was a non-conservative segment behind the catalytic domain (TIM barrel domain) of *At*GUS which showed low identity with *P*GUS and *Au*GUS after the primary sequence alignment. A model of the three-dimensional structure of *At*GUS was presented in the current research (**Figure 7**). The deleted sequence exhibited no involvement in the TIM barrel domain, locating near the “stability face” rather than the “catalytic face” [21].

*At*GUS-E and *At*GUS(-3t)-E were very similar with each other at some enzymatic properties, such as optimal pH and optimal temperature. It was reported in previous studies that many modified enzymes maintained some original enzymatic properties even though some sequence has been modulated [10]. Furthermore, we could also speculate that the non-conservative sequence lied outside of the catalytic face of the TIM barrel domain which may not affect the catalytically active residues and the GL biotransformation mode.

Interestingly, the stability of *At*GUS(-3t)-E was slightly higher than that of *At*GUS-E at 65°C. Similar results have been reported that the modification of the C-terminal region could improve the thermal stability of endo-β-glucanase from *Bacillus subtilis* JA18 [11]. In addition, previous study showed that αβ-loops in “stability face” are important for the stability [22]. The truncation of the non-conservative sequence lies near αβ-loops of the stability face, therefore, we can predicted that the deletion of the C-terminal region outside the TIM barrel domain has influence on thermal stability of *At*GUS.

The effect of nine metal ions with different concentration gradients on the activities of *At*GUS(-3t)-E and *At*GUS-E was evaluated, and Co^2+^, Ca^2+^, and Ni^2+^ showed opposite effects on the two enzymes, respectively. In addition, *At*GUS(-3t)-E showed higher affinity and catalytic efficiency than *At*GUS(-3t)-E. Both of the result might suggest that the spatial structural rearrangement, and the speculation has been proved by CD spectra, which showed great difference between the secondary structure of the two enzymes. It has been reported that the loops above the catalytic face was very important for substrate hydrolysis [23], so we can conclude that the truncation of the non-conservative domain firstly changed the secondary structure of the enzyme and then influenced the substrate affinity, catalytic efficiency and metal ions effects. Moreover, the crystal structure of human β-glucuronidase was firstly reported in 1996 [24], and the structure of bacterial β-glucuronidase has also been published recently [25]. Both β-glucuronidase structures were tetramers. The deleted region of the *At*GUS non-conservative sequence lies outside the main three domains, so it was predicted that the non-conservative might change the combination pattern of each monomer.

Different methods have been applied in creating new enzyme such as error-prone PCR [26], DNA shuffling [27] and staggered extension process (StEP) [28]. Some efforts have been made to modify the enzyme by directed screening but high ratio of negative mutated forms of enzyme in the initial screening is a big hurdle and requires further screening for positive mutated forms of enzyme. Every method has its own advantages and disadvantages that determines the feasibility of a particular method so as sequence truncation [10], [11], [12]. Based on the same hydrolyzing mode, relatively higher thermal stability, and especially the enhanced affinity and catalytic efficiency for GL, deletion of the non-conservative sequence behind the TIM barrel domain was a successful evolution of *At*GUS. The truncation of non-conservative region based on sequence alignment could be an effective way of artificial enzyme evolution as it can alter and influence the stability and catalytic efficiency of enzyme, and could help in understanding the relationship between the structural modulation and enzymatic properties.
